# Calorie Restriction Decreases Murine and Human Pancreatic Tumor Cell Growth, Nuclear Factor-κB Activation, and Inflammation-Related Gene Expression in an Insulin-like Growth Factor-1−Dependent Manner

**DOI:** 10.1371/journal.pone.0094151

**Published:** 2014-05-07

**Authors:** Alison E. Harvey, Laura M. Lashinger, Drew Hays, Lauren M. Harrison, Kimberly Lewis, Susan M. Fischer, Stephen D. Hursting

**Affiliations:** 1 Department of Nutritional Sciences, University of Texas, Austin, Austin, Texas, United States of America; 2 Department of Molecular Carcinogenesis, University of Texas M.D. Anderson Cancer Center, Smithville, Texas, United States of America; Thomas Jefferson University, United States of America

## Abstract

Calorie restriction (CR) prevents obesity and has potent anticancer effects that may be mediated through its ability to reduce serum growth and inflammatory factors, particularly insulin-like growth factor (IGF)-1 and protumorigenic cytokines. IGF-1 is a nutrient-responsive growth factor that activates the inflammatory regulator nuclear factor (NF)-κB, which is linked to many types of cancers, including pancreatic cancer. We hypothesized that CR would inhibit pancreatic tumor growth through modulation of IGF-1-stimulated NF-κB activation and protumorigenic gene expression. To test this, 30 male C57BL/6 mice were randomized to either a control diet consumed ad libitum or a 30% CR diet administered in daily aliquots for 21 weeks, then were subcutaneously injected with syngeneic mouse pancreatic cancer cells (Panc02) and tumor growth was monitored for 5 weeks. Relative to controls, CR mice weighed less and had decreased serum IGF-1 levels and smaller tumors. Also, CR tumors demonstrated a 70% decrease in the expression of genes encoding the pro-inflammatory factors S100a9 and F4/80, and a 56% decrease in the macrophage chemoattractant, Ccl2. Similar CR effects on tumor growth and NF-κB-related gene expression were observed in a separate study of transplanted MiaPaCa-2 human pancreatic tumor cell growth in nude mice. *In vitro* analyses in Panc02 cells showed that IGF-1 treatment promoted NF-κB nuclear localization, increased DNA-binding of p65 and transcriptional activation, and increased expression of NF-κB downstream genes. Finally, the IGF-1-induced increase in expression of genes downstream of NF-κB (Ccdn1, Vegf, Birc5, and Ptgs2) was decreased significantly in the context of silenced p65. These findings suggest that the inhibitory effects of CR on Panc02 pancreatic tumor growth are associated with reduced IGF-1-dependent NF-κB activation.

## Introduction

Pancreatic cancer is the fourth leading cause of cancer-related death in the United States [1], with a 5-year survival rate of only 3–5% [2]. Recent epidemiological studies suggest that obesity is associated with a 2-fold higher risk for pancreatic cancer in both males and females [3]. Moreover, 10 prospective cohort studies also report an increased risk for pancreatic cancer for obese individuals with a body mass index (BMI) of >30 compared to healthy weight individuals with a BMI of <25 kg/m_2_ individuals [4]. The connection between obesity and increased cancer risk is attributed to the myriad of metabolic perturbations associated with increased adiposity, although the specific contribution of each is largely unknown. Included in the effects of obesity is its association with a chronic low-grade state of inflammation as well as elevations in blood glucose and alterations in serum factors such as insulin, leptin, adiponectin, and insulin-like-growth factor (IGF)-1 [5]. Together these circulating factors provide a complex signaling network which functions to regulate metabolism and are hypothesized to be involved with several aspects of tumor development.

Obesity-related inflammation occurs predominantly in adipose tissue and is characterized by elevated expression and production of pro-inflammatory chemokines and cytokines such as monocyte chemoattractant protein (MCP)-1, interleukin (IL)-6, and interleukin (IL)-1β. The obese inflammatory environment also consists of infiltrating immune cells, particularly macrophages that function in an autocrine and paracrine manner to perpetuate the inflammatory process through cytokine production and prostaglandin synthesis catalyzed by the inflammatory enzyme cyclooxygenase (COX)-2 [6]. Susceptibility to pancreatic cancer is largely influenced by chronic inflammation, as evidenced by the strong association between pancreatitis and pancreatic cancer risk [7]. Biopsies taken from patients with chronic pancreatitis show increased protein expression of monocyte chemoattractants and macrophage infiltrates compared to normal pancreatic tissue [8]. Moreover, tumors taken from pancreatic cancer patients demonstrate elevated pro-inflammatory gene and protein expression of IL-6, IL-1β, and COX-2, as well as increased expression of monocyte chemoattractants [9], [10], [11].

Inflammation is mediated through multiple pathways, one of which is the nuclear factor kappa-light-chain-enhancer of activated B-cells (NF-κB) pathway. NF-κB is a transcription factor that is activated in response to various stimuli including growth factors and inflammatory molecules, and is responsible for inducing gene expression associated with cell proliferation, apoptosis, angiogenesis, and inflammation [12]. NF-κB is upregulated in over 70% of human pancreatic cancer cell lines and primary tumors [13] and has been linked with increased angiogenic, invasive, and metastatic capability in pancreatic cancer cells [14], [15], [16]. Furthermore, elevated circulating levels of energy balance-related hormones, such as leptin, insulin, and IGF-1 have been shown to activate NF-κB signaling through upregulation of the PI3K/Akt/mammalian target of rapamycin (mTOR) pathway [17]. Activation of NF-κB by mTOR pathway activation leads to the phosphorylation of the NF-κB inhibitory protein, IκBα, which is targeted for ubiquitination by the 26S proteasome allowing the active NF-κB subunit, p65, to translocate to the nucleus and intiate transcription of multiple genes associated with inflammation and cancer development [18], [19]. The impact of NF-κB signaling is compounded by the fact that downstream gene products, such as IL-6, COX-2, and IL-1β, amplify the inflammatory environment through direct and indirect mechanisms [20].

Calorie restriction (CR), an effective anti-obesity strategy with potent anticancer effects, has been shown to reduce serum levels of insulin, leptin, and IGF-1 and decrease the expression of protumorigenic cytokines [21], [22]. The anticancer effects of CR have been observed in many cancers [23], and as little as 10% CR has been shown to have a growth inhibitory effect in a chemically-induced model of pancreatic cancer [24]. We have shown that 30% CR significantly decreases incidence of pancreatitis-induced and Kras-induced pancreatic cancer in genetically engineered mouse models (GEMMs), largely through reduction of circulating levels of IGF-1 [25], [26], [27]. In those reports, we focused on the impact of CR on reduced pancreatic cancer cell proliferation via decreased serum IGF-1 levels and decreased signaling through the mTOR pathway. Using genetic and pharmacologic approaches, we found that mTOR pathway inhibition explains only ∼40–50% of the anticancer effects of CR in our GEMMs. We focus in this report on the link between reduced IGF-1 levels in CR mice and reduced inflammatory signaling, which we and others have found to be an important contributor to the anticancer effects of CR in varied model systems [28], [29]. Four key findings specifically suggest a strong interplay between CR, IGF-1 and NF-κB. First, our previous studies in GEMMs of pancreatic cancer showed that CR substantially decreases the reactive stroma (fibrosis and inflammatory cell infiltration) associated with progressive pancreatic lesions in our BK5.COX-2 mouse model of pancreatitis-induced pancreatic cancer [27]. CR has also been shown to both offset the age-induced activation of NF-κB in HEK293T cells and aged kidney [30], [31], and to decrease production of serum inflammatory markers [32]. In a murine colon cancer model, CR reduces tumor burden through IGF-1–dependent reductions in NF-κB signaling [28]. Finally, genetic reduction of circulating IGF-1 to levels observed in CR mice results in decreased expression of a broad panel of inflammatory cytokines known to be regulated by NF-κB [33]. Thus, in the present study we investigated whether a) the growth-inhibitory effects of CR in the Panc02 tumor model could be linked via reduced IGF-1 levels to a reduction in NF-κB activity and subsequent reduction of pro-inflammatory mediators and b) the elevated levels of circulating IGF-1 observed in response to positive energy balance could enhance activity of NF-κB.

## Materials and Methods

### Mice, diets, and study design

All animal experimentation was conducted in strict accordance with the recommendations outlined in the Guide for the Care and Use of Laboratory Animals set forth by the National Institutes of Health. The corresponding animal protocol was approved by the Institutional Animal Care and Use Committee of the University of Texas (#2009-0073). All efforts were taken to minimize animal suffering.

#### Panc02 murine pancreatic tumor cell transplant study

Thirty 6-week old male C57/BL6 mice were purchased from Charles River Laboratories (Frederick, MD) and were singly housed in a semibarrier facility at the University of Texas at Austin Animal Resource Center. All mice were fed a chow diet (Harlan Diets, catalog #2018, Madison, WI) for one week after arrival and then randomly assigned to receive one of two diets for 26 weeks (n = 15/group): 1) control diet (#D12450B, a modified AIN-76A semipurified diet with 10 kcal% fat, consumed ad libitum) which generates an overweight phenotype or 2) 30% calorie restriction (CR, #D0302702), which generates a lean phenotype. The CR diet was a modified AIN-76A semipurified diet that when fed as a daily aliquot, provided 70% of the total caloric intake but 100% of all vitamins, minerals, fatty acids and amino acids consumed by the control mice. Diets were purchased from Research Diets (New Brunswick, NJ). Mean body weight per group was recorded through 20 weeks (1 week prior to tumor cell injection). At week 21, body composition (fat mass, lean mass, % body fat) was measured using quantitative magnetic resonance (Echo Medical Systems, Houston, TX) and mice underwent isoflurane anesthesia and retro-orbital venipuncture for blood collection. Whole blood was centrifuged at 10,000×g for 5 minutes and serum was removed and stored at −80°C for subsequent analyses. After 21 weeks on diet, mice were injected subcutaneously with 5×10^5^ Panc02 murine pancreatic adenocarcinoma cells (generously provided by Dr. J. Schlom, NCI, ref [34]) into the right flank, then continued on their diet regimens, and tumor growth was monitored for an additional 5 weeks. Tumors were palpated and measured with Vermeer calipers weekly, and tumor volume was calculated using the formula 4/3∏ (r_1_)^2^(r_2_). After 5 weeks of tumor growth, mice were fasted for 12 hours, anesthetized with isoflurane for blood collection by cardiac puncture then killed by cervical dislocation. Tumors were excised and weighed, then either flash frozen in liquid nitrogen and stored at −80°C or fixed in 10% neutral-buffered formalin overnight, then switched to ethanol until they were processed and paraffin embedded.

In preparation for injection, Panc02 cells, derived from a chemically induced grade II adenocarcinoma (Corbett et al, 1984), were maintained in a 37°C incubator under an atmosphere of 5% CO_2_ with high glucose McCoy's 5A Modified Media (Invitrogen, Carlsbad, CA), supplemented with 10% fetal bovine serum (FBS; Hyclone), 2 mM glutamine (Invitrogen), 10,000 U/mL penicillin, 10,000 U/mL of streptomycin, 1X Non Essential Amino Acids, 10 mM HEPES buffer, and 1 mM sodium pyruvate.

#### MiaPaCa-2 human pancreatic tumor cell transplant study

Thirty 6-week old male athymic nude mice were purchased from Jackson Laboratories, (Bar Harbor, ME) and placed on chow diet for 1 week following arrival at the UT Austin Animal Resource Center. Mice (n = 15/group) were randomized to receive either the control diet or 30% CR diet used in the previous study. Animals were singly housed and received diet for 23 weeks. Mean body weight per diet group was recorded for 16 weeks (1 week prior to tumor cell injection). At week 17 of diet treatment, mice were subcutaneously injected with 4×10^6^ MiaPaCa-2 human pancreatic cancer cells (American Type Culture Collection (ATCC), Manassas, VA) in the right flank. Tumor growth was monitored and recorded as previously described for 6 more weeks. Mice were then fasted for 12 hours, anesthetized with isoflurane then killed by cervical dislocation. Tumors were excised and weighed then prepared as previously described. Whole blood was also harvested and prepared as previously described. One mouse from each group did not develop any tumors so were excluded from analysis, thus final sample size is 14.

Prior to injection, the MiaPaCa-2 cells were maintained in DMEM with 10,000 U/mL penicillin, 10,000 U/mL of streptomycin, 1X Non Essential Amino Acids, 10 mM HEPES buffer, and 1 mM sodium pyruvate.

### Serum marker analyses

For the Panc02 transplant study, blood was collected after 21 weeks on diet and analyzed for circulating levels of insulin, leptin, adiponectin, and MCP-1, which were measured using the Lincoplex bead-based multiplexed assays (Millipore Corporation, Billerica, MA) on a BioRad Bioplex 200 analyzer (BioRad, Hercules, CA) according to manufacturer's directions. Serum IGF-1 levels were also analyzed at this time point by ELISA per manufacturer's instructions (Quantikine MG-100, R&D Systems, Minneapolis, MN). Sample size for analysis of each analyte was eight randomly selected mice per group. At time of study endpoint (26 weeks on diet), fasting blood glucose was measured on terminal blood samples using a Contour glucometer with Contour glucose test strips (Bayer HealthCare LLC, Mishawaka, IN Glucometer).

### Real-time RT-PCR analyses of inflammatory, angiogenesis and cell cycle-related gene expression

Total RNA was extracted from Panc02 and MiaPaCa-2 tumors using the Qiagen RNeasy Mini Kit according to the manufacturer's protocol (Qiagen, CA). A total of 2 µg of RNA was reversed transcribed using High-capacity cDNA Reverse Transcription kits (Ambion, Austin, TX). Expression of inflammatory, angiogenic, and cell cycle-related genes including F4/80, chemokine (C-C motif) ligand (CCL) 2, S100A9, Ccdn1, Vegfa, Birc5, Ptgs2, IL-6, RELA (p65 subunit), and XIAP was determined through real-time PCR using TaqMan primer-probes (Ambion, Austin, TX). Gene expression was normalized to the housekeeping gene, β-actin and analyzed using the ΔΔCT method.

### Immunohistochemical staining of phospho-p65 (p-p65)

Formalin-fixed tissue was embedded in paraffin and cut into 4 µm thick sections for immunohistochemical analysis. Slides were deparaffinized in xylene and sequentially rehydrated in ethanol to water. Endogenous peroxidase activity was blocked by incubating slides with 3% H_2_O_2_ for 10 minutes. Antigen retrieval was performed with 10 mM citrate buffer for 15 minutes in a microwave oven and cooled down for 20 minutes. Nonspecific antibody binding was inhibited by incubating slides for 10 minutes with Biocare Blocking Reagent (Biocare, Concord, CA). Slides were then incubated with primary p-p65 antibody (Cell Signaling, Boston, MA (mouse), Santa Cruz Biotechnology, Santa Cruz CA (human) at a 1∶100 dilution overnight at 4°C. Slides were then incubated with Dako EnVision-labeled polymer, anti-rabbit-HRP (Dako, Carpinteria, CA) for 30 minutes at room temperature, followed by incubation with Dako diaminobenzidine and counterstained with hematoxylin. Images were captured using a light microscope equipped with a Leica digital color camera (Leica Camera Inc, Allendale, NJ) and incubated in SA-HRP (BioGenex, Fremont, CA) for 30 minutes at room temperature. The number of p-p65-positive cells that displayed either nuclear or cytoplasmic localization was determined using 40X images of 10 and 7 tumors from the control and CR groups, respectively; at least 3 fields per tumor were analyzed. Sample size was limited by the availability of paraffin-embedded tumor blocks.

### Cytokine and chemokine analyses in supernatants from IGF-1-treated cells

Panc02 cells were seeded in 6 well plates in 10% FBS-supplemented media for 24 hours. Following a four-hour serum starvation, cells were treated with IGF-1 (400 ng/mL; R&D Systems) for 24 hours then supernatant was collected and stored at −20°C until the assay was performed. Levels of protein expression were analyzed using the Lincoplex bead-based cytokine/chemokine panel (Millipore Corporation, Billerica, MS) on a BioRad Bioplex 200 analyzer (BioRad) according to manufacturer's directions. Choice of concentration (400 ng/mL) was based on physiologically relevant levels of circulating IGF-1 observed in mice fed the same control diet used in the present study [35], [36], [37]. Supernatants were collected from three separate experiments.

### Immunofluorescence of p65

Approximately 1×10^5^ Panc02 cells were seeded on chamber slides (Lab-Tek, Rochester, NY), allowed to grow for 24 hours, then serum starved for 4 hours and treated with IGF-1 (400 ng/mL) for 30 minutes, 1 hour, or 4 hours in serum-free media. Cells were then fixed in 4% paraformaldehyde/1x PBS for 10 minutes at room temperature, washed in 1x PBS, permeabilized in 1x PBS/0.1% triton X-100 (PBST) for 2 minutes, and washed again in 1x PBS. Formaldehyde cross-linking was neutralized in 1x PBS/100 mM glycine for 5 minutes at room temperature and cells were washed in 1x PBS. Primary antibody was added (p65, 1∶300; Cell Signaling) in blocking buffer (5% donkey serum in PBST) for 45 minutes at 37°C, slides were washed three times in PBST for 5 minutes at room temperature followed by staining with donkey anti-rabbit secondary antibody conjugated to fluorescein isothiocyanate (1∶300; Jackson ImmunoResearch Laboratories, Westgrove, PA) for 45 minutes at 37°C. Following three washes in PBST for 5 minutes at room temperature, slides were counterstained with 300 nM 4′-6-diamidino-2-phenylindole for 5 minutes at room temperature. Slides were mounted with ProLong Gold Antifade Reagent (Invitrogen, Carlsbad, CA). Slides were viewed on a Zeis Axiovert 200 M microscope with equal exposure at 60X oil magnifications with appropriate filter. Images shown are representative of three separate experiments.

### NF-κB DNA Binding Assay

Approximately 1×10^6^ Panc02 cells were seeded in 10 cm dishes as described above. Cells were allowed to grow in the standard DMEM+10% FBS media for 24 hours, then were serum starved for 4 hours. Following serum starvation, cells were treated with DMEM plus 10% FBS, serum-free DMEM, or serum-free DMEM+IGF-1 (400 ng/mL) for 4, 8, and 16 hours (data not shown), and protein was harvested. Briefly, attached cells were washed and collected in 1x PBS/Phosphatase inhibitors (Active Motif, Carlsbad, CA) and centrifuged at 500×g at 4°C. Following the removal of supernatant, the cell pellet was resuspended in approximately 60–100 µl of protein lysis buffer (RIPA buffer, Phosphatase Inhibitor II and III, Sigma; Roche MiniTab), kept on ice for 30 minutes with gentle agitation every 10 minutes, and centrifuged at 16,000×g for 10 minutes at 4°C. Whole cell protein extracts were quantitated by Bradford assay (Bio-Rad; Hercules, CA) and normalized to bovine serum albumin (BSA, 1 mg/mL).

A TransAM enzyme-linked immunosorbent assay (ELISA) was used to measure activation of the NF-κB subunit, p65 according to manufacturer's instructions (Active Motif, Carlsbad, CA). Briefly, 20 µg of whole cell protein lysate was diluted in lysis buffer (as described above) and loaded in duplicate onto a 96-well plate coated with an oligonucleotide containing the NF-κB consensus site (5′-GGGACTTTCC-3′). NF-κB/p65 primary antibody was added to each well and used to detect an epitope of NF-κB accessible only when activated and bound to its target DNA. An HRP-conjugated secondary antibody was added to generate a colorimetric reaction that was detected by spectrophotometry at 450 nm with a reference wavelength of 655 nm. Assays were performed on three separate experiments.

### NF-κB transcriptional activation

Approximately 1.8×10^5^ Panc02 cells were seeded in 6-well plates and allowed to attach overnight at 37°C. Twenty-four hours later, cells were transfected with pNF-κB/Luc and pRenilla/Luc (generously provided by Dr. Linda DeGraffenried) using FuGENE6 (Roche Applied Science, Indianapolis, IN). Cells were incubated with transfection agent for another 24 hours and then serum starved for 4 hours. Following serum starvation, cells were treated with McCoy's plus FBS, serum-free McCoy's, or IGF-1 (400 ng/mL) for 6 hours and protein was harvested using Dual Luciferase Reporter Assay System (Promega, Madison, WI) according to manufacturer's instructions. Assays were performed on three separate experiments.

### Small interfering RNA constructs and transfections

NF-κB silencing was accomplished by combining siRNA constructs targeting either p65 or a scrambled sequence (Ambion, Austin, TX) with Opti-MEM reduced serum media (Invitrogen; Carlsbad, CA) and siPORT Amine transfection reagent (Ambion, Austin, TX). The transfection complex was added to cells in a 6-well plate and allowed to incubate overnight. Twenty-four hours later, media containing the transfection complex was removed and cells were serum-starved for 4 hours. Following serum-starvation, cells were treated with McCoy's supplemented with 10% FBS, serum-free McCoy's, or 400 ng/mL of IGF-1 for 18 hours and RNA was harvested for NF-κB downstream gene analysis (as described previously).

### Statistics

Body weight, body fat, blood glucose, tumor volume and weight data are presented as mean ± SD, while serum hormone, PCR, supernatant protein expression, ELISA, and reporter assay data are shown as mean ± SEM. Differences in outcomes were analyzed by Student's t-test, except differences in Panc02 gene expression following IGF-1 treatment with silencing constructs, which were analyzed by one-way ANOVA followed by Tukey's post hoc test. Differences in Vegf were assessed by Tamhane's T2 as a post hoc test because of unequal variance). Data was considered significant if p<0.05.

## Results

### Body composition, blood glucose, and serum hormones/growth factors in C57BL/6 mice following 21 weeks of dietary energy balance modulation

**Figure 1:**
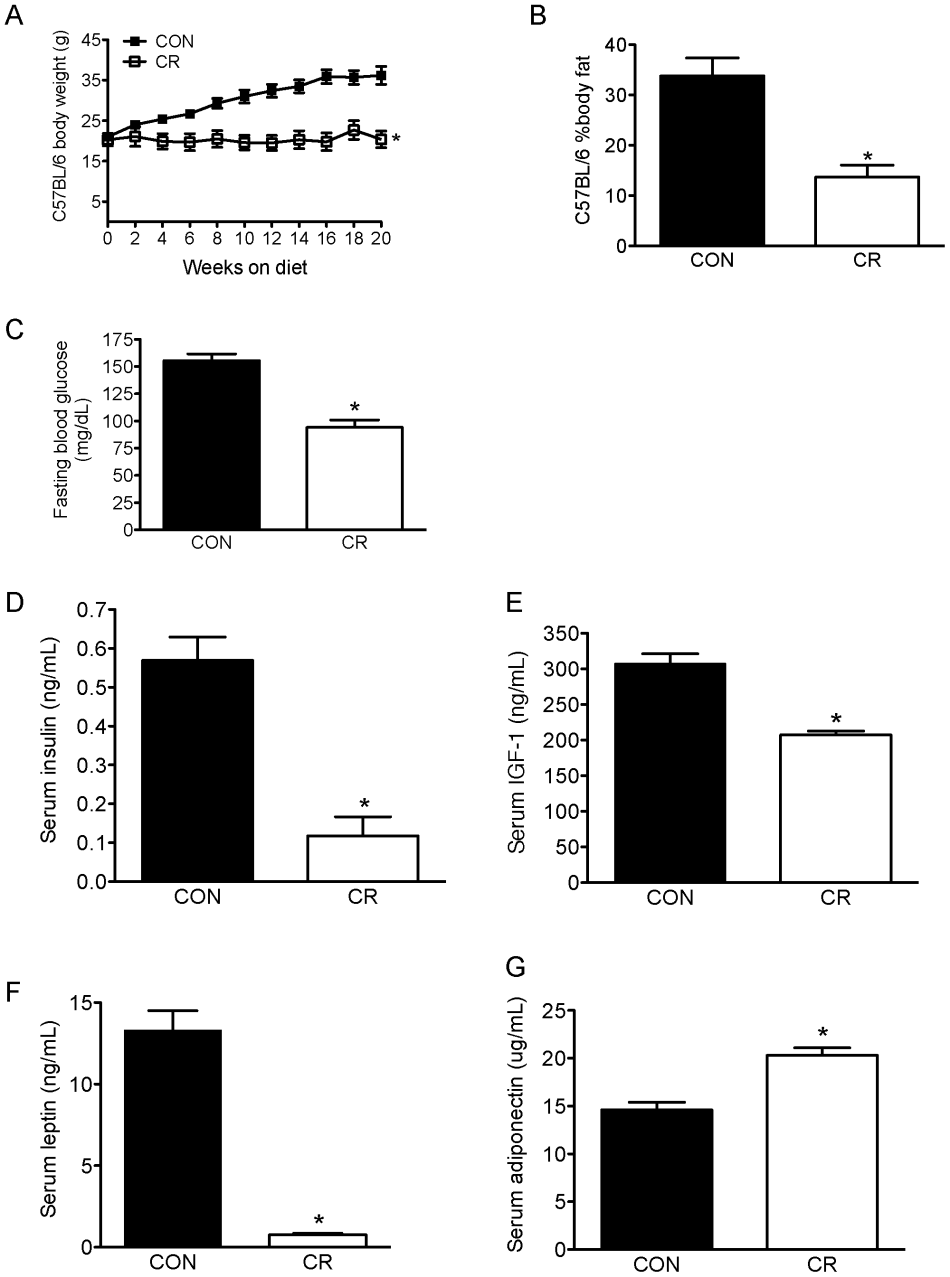
Body composition, blood glucose, and serum factors in C57BL/6 mice following 21 weeks of diet treatment. Calorie restriction (CR) decreased A, body weight (reported up to 20 weeks on diets, before tumor cell injection); B, percent body fat (at 21 weeks on diet); and C, fasting blood glucose (at 21 weeks on diet) relative to CON diet (n = 15/group). Data shown represent mean ± SD. CR altered mean fasting serum levels of D, insulin; E, IGF-1; F, leptin; and G, adiponectin (n = 8/group). Data shown represent mean ± SEM. * denotes significant differences between CR and CON. CR, calorie restriction; CON, control diet; IGF-1, insulin-like growth factor-1.

The two dietary interventions generated different weight phenotypes in C57BL/6 mice. After 20 weeks on study, CR mice weighed significantly less (20.4 g±2.1 g, p<0.05) than control mice (36.0 g±2.2 g; Fig. 1A). This weight difference was directly proportional to the levels of adiposity, with CR having a significantly lower percent body fat (14%±2%, p<0.05) than control mice (34%±4%; Fig. 1B), and fasting blood glucose (p<0.0001; Fig. 1C).

Serum analysis of energy balance-responsive hormone and growth factor levels revealed that following 21 weeks of diet treatment, fasting insulin (Fig. 1D), IGF-1 (Fig. 1E), and leptin (Fig. 1F) levels were lower in CR mice compared to control (p<0.05 for each). Conversely, levels of adiponectin increased in response to the CR regimen relative to control diet (p<0.05; Fig. 1G).

### CR reduced Panc02 tumor burden and inflammatory profile

**Figure 2:**
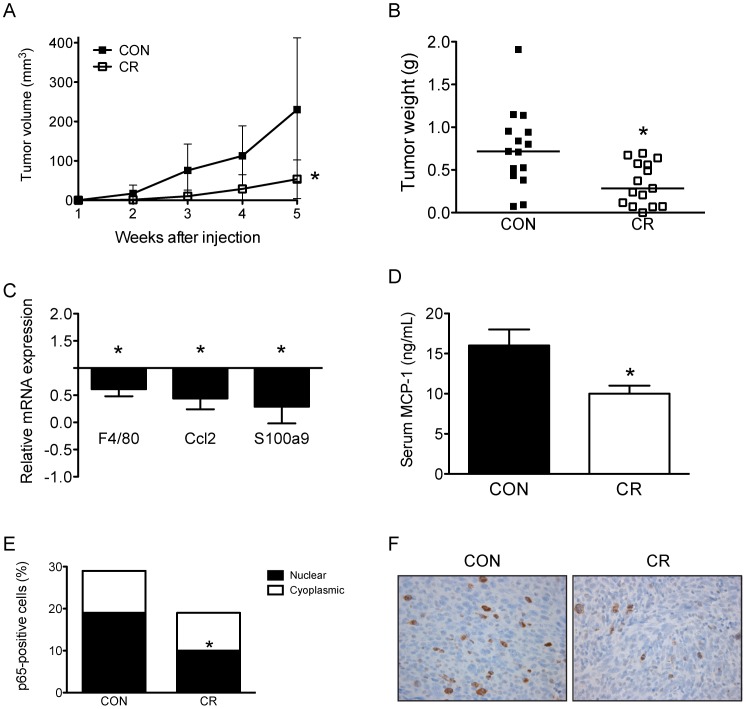
CR reduced Panc02 tumor volume and inflammatory profile. CR decreased *A*, tumor growth and *B*, median tumor weight compared to CON mice (n = 15/group). Data shown are mean ± SD with the exception of the line in scatter plot representing the median tumor weight. *C*, CR reduced mRNA expression within the tumor microenvironment compared to CON mice (CON, n = 6; CR, n = 5). Data represent CR gene expression relative to CON. *D*, Mean fasting serum levels of MCP-1 (n = 8/group, randomly selected). *E*, Representative micrographs of p-p65-staining tumor sections (40X). *F*, Quantification of staining. Bar graph represents total percentage of p-p65-positive cells, with further illustration of intracellular localization (nuclear, black; cytoplasmic, white) (CON, n = 10 and CR, n = 7). Data shown represent mean ± SE. * denotes significant differences between CR and CON. CR, calorie restriction; CON, control diet; MCP-1, monocyte chemoattractant factor-1; p-p65, phosphorylated p65.

C57BL/6 mice were subcutaneously injected with Panc02 pancreatic tumor cells after being on their diet regimens for 21 weeks. Final tumor volumes were significantly smaller in CR mice (p<0.05) relative to control mice (Fig. 2A) as were the *ex vivo* tumor weights (p<0.05; Fig. 2B).

Analysis of inflammatory gene expression showed that tumors from CR mice, relative to controls (n = 3 mice/group), demonstrated a 70% decrease in the expression of genes encoding the inflammatory markers, F4/80 and S100a9 (p<0.05 for each; Fig. 2C) and a 56% decrease in the expression of Ccl2 (p<0.05; Fig. 2C), the gene encoding the macrophage chemoattractant MCP-1. Furthermore, we found that circulating levels of MCP-1 were significantly lower in the CR mice than the control mice (p<0.05; Fig. 2D). To determine the level of *in vivo* activation of NF-κB in the tumor, sections were stained with an antibody specific for phosphorylated p65 and cells positive for nuclear versus cytoplasmic localization were counted. Tumors from CR mice had significantly fewer cells with nuclear p65 localization relative to the control tumors (p<0.01; Fig. 2E&F) although the total number of p-p65-positively stained cells was not different between the diet groups (Fig. 2E).

### IGF-1 increased p65 nuclear localization and activity

**Figure 3:**
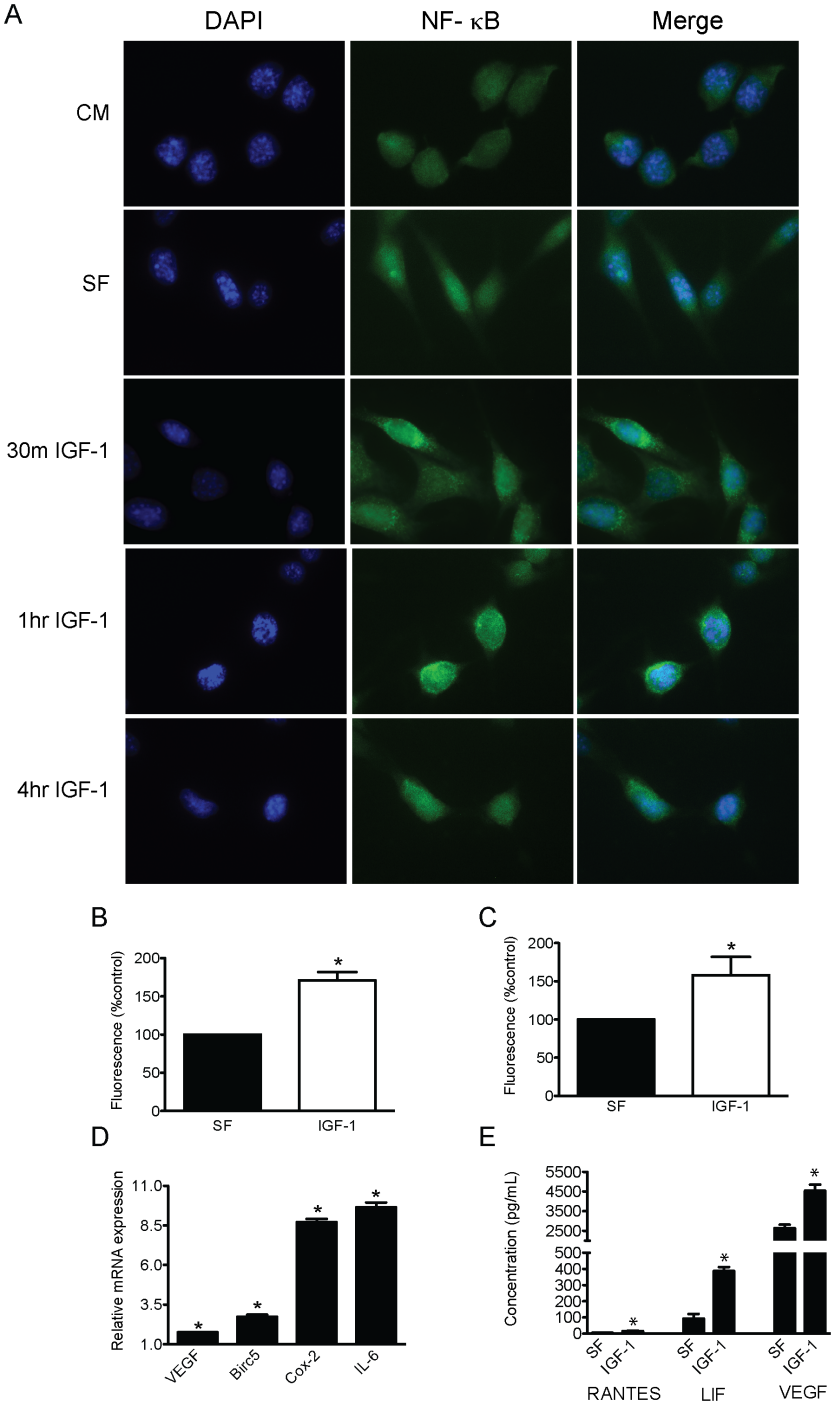
Effect of IGF-1 on p65 nuclear localization and NF-κB pathway activation in Panc02 cells. A, Representative fluorescent images of p65 localization at various time points following IGF-1 treatment (400 ng/mL) using 300 nM DAPI alone, a p65 antibody alone (p65), or p65 antibody counterstained with DAPI (merged). Data representative of 4 separate assays. B, ELISA of p65 DNA binding in response to 4-hour IGF-1 treatment (400 ng/mL). C, NF-κB luciferase reporter assay after 6-hour IGF-1 treatment (400 ng/mL). *denotes significant differences between SF and IGF-1. D, PCR analysis of mRNA expression of NF-κB downstream gene targets after 18-hour IGF-1 treatment. E, ELISA measurement of RANTES, LIF, and VEGF (n = 3) expression in Panc02 supernatant after 24 hours of IGF-1 treatment. *denotes significant differences between SF and IGF-1 with respect to each respective gene or protein of interest. All experiments used 400 ng/mL of IGF-1. Bar graphs represent mean ± SEM (n = 3 separate experiments performed in triplicate for B, C, and D). SF, serum-free; IGF-1, insulin-like growth factor-1; DAPI, 4′-6-diamidino-2-phenylindole; RANTES, Regulated on Activation, Normal T cell Expressed and Secreted; LIF, leukemia inhibitory factor; VEGF, vascular endothelial growth factor.

Nuclear localization of the p65 subunit in Panc02 cells was assessed in response to IGF-1 treatment (400 ng/mL). Translocation of p65 occurred as early as 30 minutes of treatment, increased at 1 hour, and peaked at 4 hours (Fig. 3A). In addition, IGF-1 treatment increased DNA binding of p65 at 4 hours (p<0.05; Fig. 3B) with no differences observed at 8 or 16 hours (data not shown). We used a luciferase reporter assay to determine the ability of IGF-1 to activate the NF-κB transcriptional machinery. Following treatment of Panc02 cells with 400 ng/mL of IGF-1 for 6 hours, luciferase activity in Panc02 cells was significantly increased compared to serum-free control (p<0.05; Fig. 3C). Beyond this time point there was no additional effect on luciferase activity (data not shown). Moreover, several genes downstream of NF-κB were upregulated, including the inflammatory-responsive genes *Ptgs2* (encodes COX-2) and *IL-6*, which were increased up to 9-fold and 10-fold, respectively; the antiapoptotic gene, *Birc5* (which encodes survivin), which was increased 2.5-fold; and the proangiogenic gene *Vegfa*, which was increased 1.7-fold (p<0.05 for each, Fig. 3D). To investigate the impact of IGF-1 on secreted biomarkers whose genes are under NF-κB regulation, Panc02 cells were treated with 400 ng/mL of IGF-1 for 24 hours and supernatant levels of RANTES, LIF, and VEGF were assessed. Indeed, IGF-1 at this concentration significantly increased expression of all three NF-κB-related proteins relative to their respective serum-free controls (p<0.01 for each analyte; Fig. 3E).

### Silencing p65 expression minimizes IGF-1-induced gene expression

**Figure 4:**
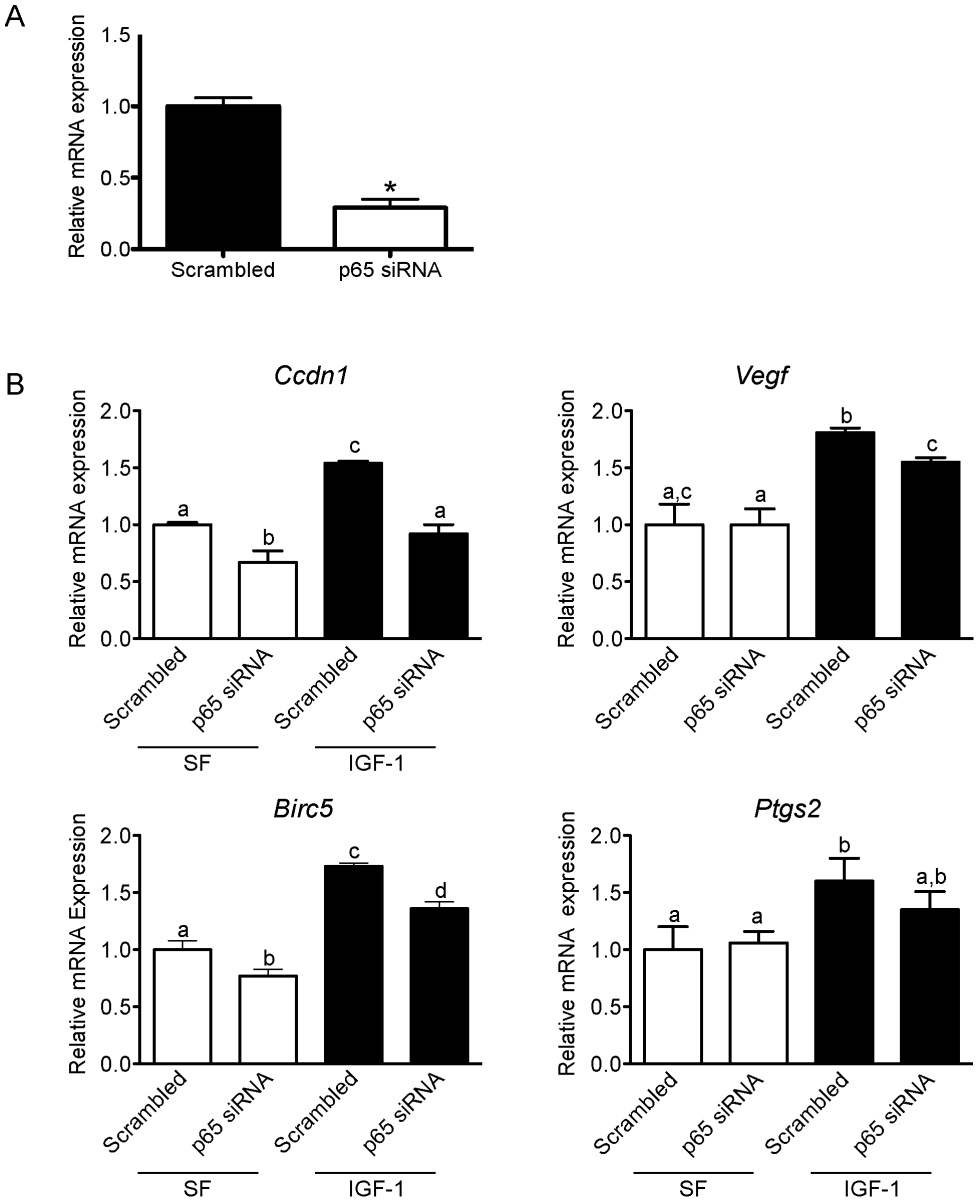
Effect of silenced p65 on IGF-1-induced expression of downstream NF-κB targets in Panc02 cells. A, Expression of p65 mRNA in Panc02 cells after exposure to either scrambled or p65-specific small interfering RNA. B, Ccdn1; Vegfa; Birc5; and COX-2 mRNA expression after 18-hour IGF-1 treatment (400 ng/mL). Bar graphs represent mean ± SEM (n = 3 separate experiments performed in triplicate for each gene). Different letters or asterisk denotes significance between groups. IGF-1, Insulin-like growth factor-1.

Transfection of Panc02 cells with siRNA specific to p65 decreased p65-induced gene expression by 70–80% within 48 hours (p<0.05; Fig. 4A). Silencing p65 reduced baseline expression (in serum-free conditions for 18 hours) of *Ccdn1* and *Birc5* (p<0.01 for each), but not *Vegf* and *Ptgs2*, relative to the scrambled sequence (Fig. 4B) in Panc02 cells. IGF-1 treatment for 18 hours (relative to serum-free controls) increased expression of NF-κB downstream genes when transfected with nonspecific scrambled control siRNA (*Ccdn1*: 54% (p<0.0001), *Vegfa*: 81% (p<0.05), *Birc5*: 73% (p<0.0001), and *Ptgs2*: 60% p<0.05; Fig. 4B). However, when p65 was silenced (compared to the scrambled sequence), the effect of IGF-1 treatment on *Ccdn1, Vegf, Ptgs2* and *Birc5* expression was blunted.

### CR reduced MiaPaCa-2 body weight, tumor burden and inflammatory gene expression

**Figure 5:**
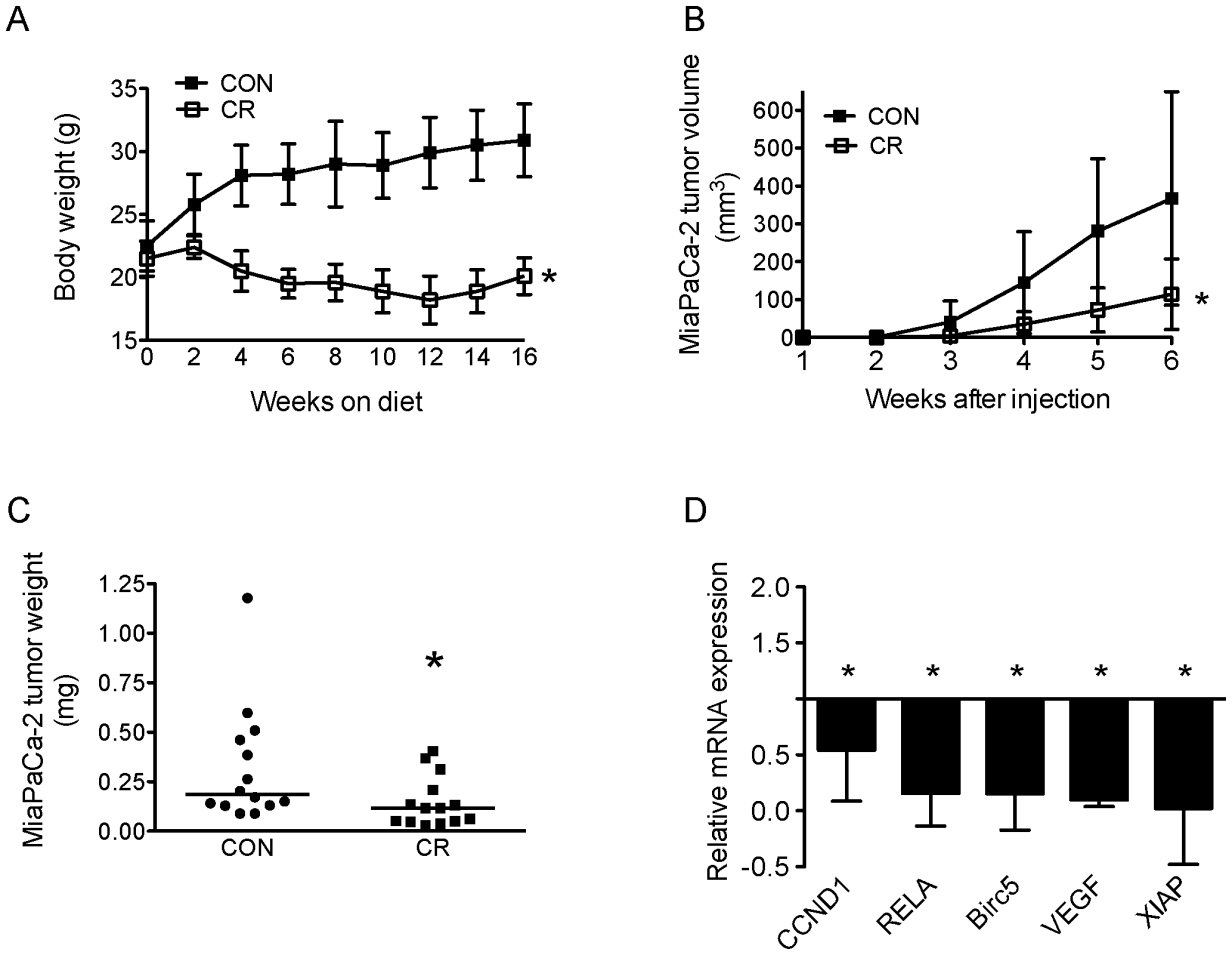
Diet effects on MiaPaCa-2 tumor burden and gene expression. A, Mean body weight of athymic nude mice per group after 16 weeks of diet treatment (prior to tumor injection). B, MiaPaCa-2 tumor volume measured over 6 weeks of tumor growth. Line graphs represent mean ± SD. C, MiaPaCa-2 tumor weight measured at study termination; line in scatter plot represents median tumor weight. A, B, & C, n = 14/group. D, Diet effects on mRNA expression of NF-κB downstream targets. Data represent CR, relative to CON, mRNA expression, mean ± SEM (n = 6/group). Significance (p<0.05) is denoted by *. CON, control; CR, calorie restriction.

Paralleling our findings in C57BL/6 mice, the control and CR diets generated two different weight phenotypes in male athymic nude mice (Fig. 5A). CR mice weighed significantly less than mice on control diet beginning after three weeks on diet (p<0.05) and continued throughout the study (p<0.05).

To test the effectiveness of energy balance modulation in a model of human pancreatic cancer cell growth, MiaPaCa-2 cells were injected into athymic nude mice after 17 weeks on diet. After 6 weeks of growth, tumors in CR mice were significantly smaller (Fig. 5B) and weighed significantly less (Fig. 5C) than tumors from control mice (p<0.05 and p = 0.05, respectively). Analysis of inflammatory gene expression revealed that tumors from CR mice, relative to controls (n = 6 mice/group), exhibited significantly lower expression of NF-κB downstream genes such as *Ccnd1* (1.8-fold decrease), *Birc5* (8-fold decrease), *VegfA* (10-fold decrease), and *Xiap* (50-fold decrease) (p<0.05 for each; Fig. 5D). Also, the expression of *RelA*, the gene encoding the p65 subunit, was decreased 6-fold (p<0.05; Fig. 5D).

## Discussion

Herein we report that CR, relative to a high calorie control diet regimen, significantly reduces body weight and body fat, modulates the nutrient-responsive serum growth factor IGF-1, decreases growth of transplanted Panc02 and MiaPaCa-2 pancreatic cancer cells in male mice, and modulates protumorigenic gene expression in Panc02 and MiaPaCa-2 tumor pancreatic tumor cells. Furthermore, levels of IGF-1 approximating those found in overweight or obese mice increase NF-κB nuclear translocation, DNA binding, transcriptional activation, and downstream gene expression of inflammation and protumorigenic genes in Panc02 pancreatic cancer cells. Although the anticancer effects of CR have been previously demonstrated in chemically-induced models of pancreatic cancer [24], [38], [39] as well as in genetically induced and transplanted models in our own lab [25], [26], [27], this report establishes an important mechanistic link between energy balance and the inflammatory microenvironment of pancreatic cancer.

The pancreas, in particular, is exquisitely sensitive to the effects of chronic inflammation, and pancreatic cancers display very high levels of reactive stroma and fibrosis that are indicative of a pro-inflammatory environment. We have previously shown that CR can dampen the inflammatory perturbations that typically coincide with pancreatic tumorigenesis [27], but we did not investigate the causal link between energy balance and inflammation. Human pancreatic cancer biopsies exhibit constitutively elevated activity of the pro-inflammatory mediator, NF-κB, inhibition of which can constrain pancreatic tumor growth [13]. The PI3K/Akt pathway, an established regulator of NF-κB activity, is governed by energy balance-responsive cues [25], [26], [27], [40]. Therefore, we tested whether CR would exert a beneficial influence on NF-κB signaling, particularly via modulation in the IGF-1/Akt pathway. Indeed, Panc02 tumors harvested from CR mice, which were significantly smaller than controls, displayed significant reductions in nuclear-located phosphorylated p65, an intracellular readout for activity of the NF-κB pathway. Moreover, we found that the macrophage chemoattractant, Ccl2 (which codes for the protein MCP-1) as well as two different macrophage markers, S100A9 and F4/80 were significantly reduced in CR tumors relative to control tumors. While F4/80 is recognized as a specific macrophage marker, S100A9 is expressed by both neutrophils and monocytes, but not resident macrophages. Macrophages located within the tumor microenvironment have been shown to be activated by NF-κB in multiple inflammatory-associated cancer models and to enhance cancer development through increased production of inflammatory molecules [41], [42], [43], [44].

Biological outcomes of IGF-1 signaling are often linked to PI3K/Akt/mTOR pathway activation, and subsequently NF-κB pathway activation [45]. In fact, Ma et al. [46] found in multiple human pancreatic cancer cell lines that IGF-1 treatment decreased phosphorylation of the tumor suppressor PTEN which lead to increased activation of PI3K/Akt and consequently, upregulation of NF-κB. In the murine Panc02 cells we found that IGF-1 increased nuclear localization of p65, DNA binding to p65, activation of NF-κB transcriptional machinery, and expression of NF-κB downstream genes which are associated with cell survival, angiogenesis, and inflammation. Moreover, with our p65 siRNA data we show that IGF-1-induced expression of select NF-κB downstream genes is dependent (at least in part) upon an intact NF-κB pathway. Secretory chemokines and cytokines whose genes are regulated by NF-κB were also significantly elevated in the supernatant of Panc02 cells treated with IGF-1. These *in vitro* data support and extend our *in vivo* tumor analysis demonstrating decreased nuclear translocation of NF-κB by CR in the pancreatic tumor microenvironment, and more specifically, that IGF-1 (at levels approximating those found in overweight/obese mice) activated NF-κB and increased transcription of genes associated with several hallmarks of cancer development.

Although mouse models of pancreatic cancer have previously identified the potent effect of CR on tumor growth [24], [25], [26], [27], we show here for the first time that human pancreatic cancer cells injected into athymic nude mice also exhibit reduced growth and downregulation of NF-κB gene targets in response to CR. In fact, the observed decrease in RelA expression in transplanted MiaPaCa-2 tumors in CR relative to control mice suggests that CR inhibits NF-κB signaling by reducing one of its key functional subunits. Our study also implicates IGF-1 as one of the links between energy balance and NF-κB signaling. Clinical studies have shown that increased bioavailable IGF-1 levels in serum of pancreatic cancer patients (relative to controls) are associated with accelerated pancreatic cancer death [47], [48]. Thus, targeting IGF-1 and NF-κB in the context of obesity and elevated bioavailable IGF-1 may be an effective strategy for mimicking the anticancer effects of CR and breaking the obesity-pancreatic cancer link.
